# ATF3 Suppresses Growth and Metastasis of Clear Cell Renal Cell Carcinoma by Deactivating EGFR/AKT/GSK3β/β-Catenin Signaling Pathway

**DOI:** 10.3389/fcell.2021.618987

**Published:** 2021-03-19

**Authors:** Shenglin Gao, Lei Gao, Simin Wang, Xiaokai Shi, Chuang Yue, Shuzhang Wei, Li Zuo, Lifeng Zhang, Xihu Qin

**Affiliations:** ^1^Department of Urology, The Affiliated Changzhou No. 2 People’s Hospital of Nanjing Medical University, Changzhou, China; ^2^Department of Urology, The Second Hospital of Hebei Medical University, Shijiazhuang, China; ^3^Changzhou Third People’s Hospital, Changzhou, China; ^4^Department of General Surgery, The Affiliated Changzhou No. 2 People’s Hospital of Nanjing Medical University, Changzhou, China

**Keywords:** clear cell renal cell carcinoma, activation transcription factor 3, growth, metastasis, GSEA

## Abstract

**Background:**

Clear cell renal cell carcinoma (ccRCC) is one of the most common malignant cancers in East Asia, with high incidence and mortality. Accumulating evidence has shown that ATF3 is associated with tumor progression.

**Methods:**

Using qPCR, the expression of ATF3 was detected in 93 patients with ccRCC, including 24 paired normal and tumor tissues, which were used to further compare ATF3 expression through western blotting and immunohistochemistry. Lentivirus was used for the overexpression or knockdown of ATF3, and the consequent alteration in function was analyzed through CCK8 assay, colony formation assay, wound healing assay, invasion assay, and flow cytometry. The potential mechanism affected by ATF3 was analyzed through gene set enrichment analysis (GSEA) and verified using western blotting, invasion assay, or immunofluorescence staining. Furthermore, a xenograft mouse model was used to assess the function of ATF3 *in vivo*.

**Results:**

ATF3 expression was significantly decreased in ccRCC compared to that in adjacent normal tissues. Through gain- and loss-of-function experiments performed in an *in vitro* assay, we found that ATF3 could regulate ccRCC cell proliferation, cycle progression, migration, and invasion. In the *in vivo* study, the xenograft mouse model revealed that ATF3 overexpression can inhibit the growth of ccRCC. Moreover, the mechanism analysis showed that suppression of ATF3 could lead to an increase the expression of β-catenin and promote β-catenin transfer to the nucleus, and might be affected by EGFR/AKT/GSK3β signaling.

**Conclusion:**

ATF3 could be utilized as an independent protective factor to inhibit the progression of ccRCC. Potential treatment strategies for ccRCC include targeting the ATF3/EGFR/AKT/GSK3β/β−catenin signaling pathway.

## Introduction

Clear cell renal cell carcinoma is one of the ten most common cancers in the world, and it accounts for 80%–90% of kidney malignancies and approximately 2%–3% of systemic malignancies (Capitanio et al., 2019). In the United States, approximately 73,820 new cases of kidney cancer and 14,770 related deaths were reported in 2019 (Siegel et al., 2019). Unfortunately, 20%–30% of patients with ccRCC present with local or distant metastasis at the first diagnosis (Chin et al., 2006), and approximately half of the patients develop metastatic lesions after surgery (Bukowski, 1997), leading to a severe impact on disease-related mortality. Therefore, further research on the mechanisms implicated in ccRCC development and progression is highly warranted to identify new therapeutic targets. Notably, it is essential to identify therapeutic targets and prognostic biomarkers that contribute to ccRCC metastasis.

Activation transcription factor 3 is a member of the transcription factor ATF/cAMP-responsive element-binding protein (CREB) family (Zhao et al., 2016).

Activation transcription factor 3 is involved in many physiological and pathological events, as it responds to a wide range of cellular stresses, including DNA damage (Yuan et al., 2013), oxidative stress (Kaneko et al., 2017), cell injury, and carcinogenic stimuli (Wang et al., 2016). According to previous reports, ATF3 plays an essential role in maintaining genome stability and promoting DNA damage response (Wei et al., 2019), and ATF3 could activate the tumor suppressor p53 to regulate the expression of its target genes (Wang et al., 2018). ATF3 has been reported to be involved in many pathological conditions, such as cancer (Gargiulo et al., 2013; Wang et al., 2018), infections/inflammation (Rosenberger et al., 2008; Bambouskova et al., 2018), diabetes (Wang et al., 2020), and ischemic injury of the heart, liver, or brain (Seijffers et al., 2007; Huang et al., 2015). Some studies have indicated that ATF3 expression is downregulated in human cancers, such as in colon cancer (Park et al., 2017), liver cancer (Xiaoyan et al., 2014), multiple myeloma (Ri, 2016), neuroblastoma (Tian et al., 2009), prostate cancer (Wang and Yan, 2016), malignant glioma (Guenzle et al., 2017), and non-small cell lung carcinoma (Bar et al., 2016), compared to its level in normal tissues. Li et al. (2016) found that ATF3 was reduced in esophageal squamous cell carcinoma (ESCC) compared with non-tumor adjacent tissues. Mechanistically, ATF3 suppressed ESCC progression via downregulating ID1. In bladder cancer, ATF3 suppresses metastasis by regulating gelsolin-mediated remodeling of the actin cytoskeleton (Yuan et al., 2013). ATF3 was a biomarker of tumor response and a reactivation of ATF3 by pracinostat was observed in the tumor response to the HDACi therapy (Sooraj et al., 2016). Although ATF3 may regulate cancer progression and metastasis in a context-dependent manner (Messmer et al., 1989; Yan and Boyd, 2006), its role in ccRCC remains unclear.

In this study, by comparing data from public databases, we found that ATF3 expression was significantly decreased in ccRCC tumor tissues. Functionally, as a tumor suppressor, ATF3 inhibited ccRCC cell growth both *in vivo* and *in vitro*. In addition, we demonstrated that ATF3 deactivated the EGFR/β-catenin signaling pathway and suppressed EMT. In conclusion, our study indicated that ATF3 could be used as a novel candidate for inhibiting the growth and metastasis of ccRCC.

## Materials and Methods

### Ethics Approval and Informed Consent

Informed consent was obtained from all subjects according to the Internal Review and Ethics Boards of The Affiliated Changzhou No. 2 People’s Hospital of Nanjing Medical University, and the project was in accordance with the Helsinki Declaration of 1964. All animal experimentation described in this study was performed in accordance with protocols approved by the Institutional Animal Care and Use Committee at Nanjing Medical University.

### Human Clinical Samples

All tumor tissues and adjacent normal tissues were obtained from the Department of Urology, The Affiliated Changzhou No. 2 People’s Hospital of Nanjing Medical University, from 2016 to 2018. IHC was performed on 4% formaldehyde-fixed tissues. Twenty-four pairs of fresh tumor tissues and corresponding peritumoral tissues were used for qRT-PCR and western blot (WB) assays.

### TCGA Database, Receiver Operating Characteristic Curve (ROC) and Gene Set Enrichment Analysis (GSEA)

R 3.6.2 was used to preprocess the microarray data. The clinical implication of ATF3 expression was obtained from The Cancer Genome Atlas (TCGA). ROC analyses were performed with MedCalc software to calculate the specificities, sensitivities, and accuracies at the best cut-off point. GSEA v3.0 was used to perform single gene set enrichment analysis.

### Cell Cultures and Transfection

The human immortalized proximal tubule epithelial cell line HKC8 and human ccRCC cell lines A498, ACHN, 786-O, OSRC-2, and Caki-1 were purchased from Cell Bank of the Chinese Academy of Sciences (Shanghai, China). All cells were cultured in DMEM (Waltham, MA, United States) supplemented with 10% fetal bovine serum (FBS) (Thermo Fisher Scientific). Cells were maintained at 37°C in 5% CO_2_. The cells were transfected with pLKO/pLKO.1-shATF3 and the transfected cells were selected using puromycin to establish stable gene-transfected cell lines.

### qRT-PCR Assay

For RNA extraction, TRIzol reagent (Invitrogen, Grand Island, NY, United States) was used to isolate total RNA. The total RNA was reverse transcribed to cDNA using a reverse transcription system (Bio-Rad, Hercules, CA, United States) according to the manufacturer’s instructions. The mRNA expression level of the gene of interest was determined through qRT-PCR using a Bio-Rad CFX96 system with SYBR green. GAPDH expression was used to normalize the expression levels of the target gene. The details of primers used in the study are shown in Supplementary Table 1.

### Western Blot Assay

Cells were lysed in radioimmunoprecipitation assay (RIPA) buffer containing phosphatase and proteinase inhibitors. After quantification and deformation, 30 μg protein was run in 10% SDS/PAGE gel and was then transferred to a PDVF membrane (Millipore, Billerica, MA, United States). After blocking for 2 h with 5% BSA at room temperature, the membrane was incubated with primary antibodies at 4°C overnight. Later, the membrane was rinsed three times and was incubated with secondary antibodies for 1 h at room temperature. Finally, protein bands were imaged using an ECL system (Thermo Fisher Scientific). In addition, the extraction of cell nuclear proteins and cytoplasmic proteins was performed using the Nuclear and Cytoplasmic Protein Extraction Kit (Wanlei) following the manufacturer’s manual. The antibodies used are listed in Supplementary Table 2.

### Immunofluorescence Staining

Cells transferred on chamber slides were fixed with 3.7% formaldehyde for 15 min at room temperature, and then PBS was used to wash the cells three times. Next, the cells were permeabilized with 0.25% Triton X-100 in PBS for 10 min. The non-specific binding sites were blocked with 5% normal goat serum in PBS for 30 min and the slides were incubated with β-catenin primary antibody (1:150, CST, United States) overnight at 4°C. Alexa Fluor^®^488 goat anti-mouse IgG and Fluor^®^594 goat anti-rabbit IgG (Millipore Inc., Billerica, MA, United States) were used as secondary antibodies. Images were obtained using an Olympus microscope (Tokyo, Japan).

### Invasion Assay and Wound-Healing Assay

Cell invasive activity was estimated using transwell plates coated with extracellular matrix gel from Corning (Corning, NY, United States). Cells (1 × 10^5^) were plated in the upper chambers containing 100 μL serum-free medium, whereas 500 μL medium containing 10% FBS was loaded into the lower chamber. After 24 h of incubation, the cells that migrated to the lower membrane surface were fixed with 4% paraformaldehyde and then stained with 0.1% crystal violet. The number of migratory cells was observed and counted under a light microscope. For the wound-healing migration assay, after the cells cultured in a 6-well plate reached 90% confluency, wounds were created by scraping the bottom of the wells with a 200 μL yellow plastic pipette tip. The wounds were observed and photographed at 0 h and 48 h.

### Cell Proliferation Assay and Colony Formation Assay

Stably transfected cells (5 × 10^3^/well) were cultured in 96-well plates. Cell viability was assessed using a CCK8. Cells were plated into 6-well plates at a density of 1 × 10^3^ cells per well. After 14 days, colonies were rinsed with PBS twice, fixed with methanol, and stained with 0.1% crystal violet, and the numbers were counted.

### Cell Cycle Analysis

The cells were harvested, washed with cold PBS, and fixed in 70% cold ethanol. Before analysis with a fluorescence-activated cell sorter (BD FACS Flow Cytometer), the cells were stained with 50 mg/L propidium iodide for 30 min.

### Animal Studies

The animal study protocol was approved by the Institutional Animal Use and Care Committee of Nanjing Medical University. For the xenograft group, mice were subcutaneously inoculated with 1 × 10^6^/100 μL OSRC-2 cells stably transfected with oeATF3 and pWPI. The implanted tumor volume in each mouse was monitored every 7 days, and tumor weight was calculated after 5 weeks when the mice were sacrificed.

### Statistical Analysis

SPSS 23.0 software (IBM, United States) was used to conduct the statistical analyses. The results of the experiments are shown as the mean ± standard deviation (SD). Differences in different groups were analyzed using Student’s *t*-test or one-way analysis of variance. Correlations between different parameters were analyzed using the Spearman rank test. A Kaplan–Meier curve was constructed to illustrate the overall survival (OS) and disease-free survival (DFS) in subgroups. *p* < 0.05 was considered statistically significant. The detailed results were ^∗^*p* < 0.05, ^∗∗^*p* < 0.01, ^∗∗∗^*p* < 0.001.

## Results

### ATF3 Is Downregulated in ccRCC and Is Negative Correlation With the Clinicopathological Prediction Trait

**FIGURE 1:**
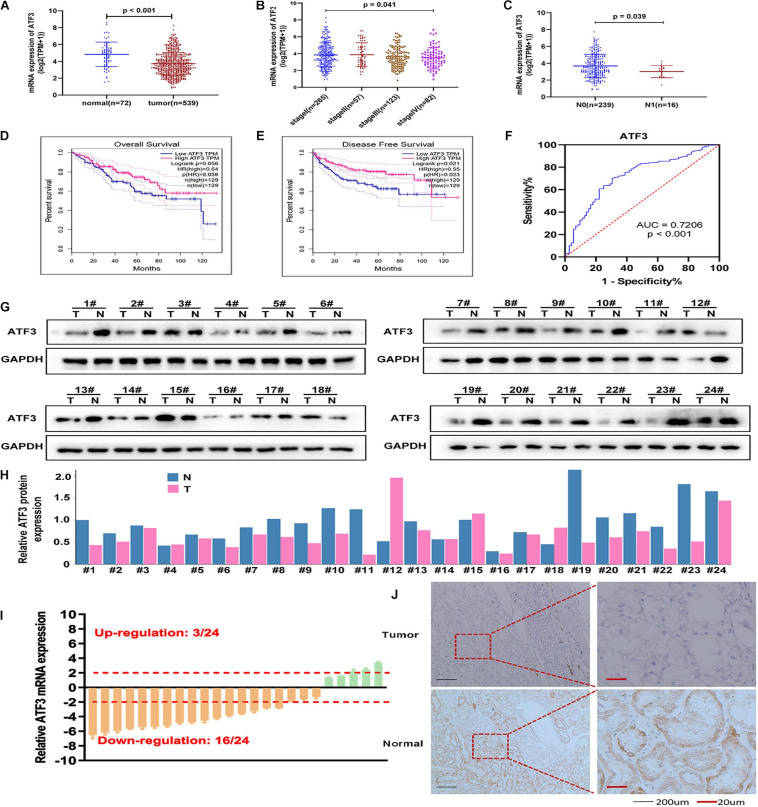
ATF3 expression decreases in ccRCC patients and acts as a protective trait. **(A)** ATF3 was downregulated in ccRCC patients compared to that in normal kidney tissues. **(B,C)** Relative expression levels of ATF3 in TCGA-KIRC subgroup: pathological stage **(B)** and lymph node metastasis **(C)**. **(D,E)** ATF3 was a positive prognostic factor for ccRCC overall survival **(D)** and disease-free survival **(E)**. **(F)** ROC curve assessed the specificity and sensitivity of ATF3 to differentiate between high and low group in ccRCC patients (AUC = 0.7206, *p* < 0.001). **(G)** Western blotting analysis of ATF3 in 24 ccRCC tissues compared to adjacent normal tissues. **(H)** The quantify of relative ATF3 protein expression. **(I)** RT-PCR analysis of ATF3 mRNA levels in 24 ccRCC tissues and adjacent normal tissues. **(J)** IHC images shown the ATF3 staining in ccRCC tissues and adjacent kidney tissues.

The expression pattern of ATF3 in ccRCC was analyzed based on RNA-seq data from the TCGA-KIRC cohort. The results showed that the mRNA expression of ATF3 was significantly downregulated in patients with RCC (*n* = 539) when compared to that in normal kidney tissues (*n* = 72) (*P* < 0.001, Figure 1A). Additionally, ATF3 expression was negatively associated with the clinical stage (*P* = 0.041, Figure 1B), lymph node metastasis (*P* = *P*-0.039, Figure 1C), poor OS (*P* = 0.056), and DFS (*P* = 0.021) (Figures 1D,E). Moreover, the area under the curve (AUC) of the ROC curve for ATF3 was 0.7206, which revealed that ATF3 could be a better prognostic signature for patients with RCC (Figure 1F). Moreover, tumor samples showed significantly lower ATF3 expression when compared with paired normal samples across 13 human tumor types (BLCA, BRCA, CHOL, HNSC, KICH, KIRC, KIRP, LIHC, LUAD, LUSC, PRAD, READ, SKCM) from TIMER web tools (Supplementary Figure 1), which further suggested that ATF3 might act as a tumor suppressor in various human cancer types. Based on ATF3 expression, the 93 patients which we have collected from the hospital were divided into high and low expression groups. As shown in Table 1, ATF3 expression was significantly related to gender, age, tumor size, Fuhrman grade, tumor stage, and metastasis in patients with ccRCC. To detect ATF3 expression at the mRNA and protein levels, we determined the expression of ATF3 in 24 pairs of ccRCC tissues and adjacent normal tissues through RT-PCR and WB assays. WB results showed that ATF3 expression was significantly downregulated in ccRCC tissues compared with that in adjacent normal tissues (Figures 1G,H), and RT-PCR assays also confirmed that there was a significant decrease in ATF3 mRNA levels in 16 of the 24 pairs of tissues (Figure 1I). Moreover, IHC staining showed that ccRCC tissues expressed lower levels of ATF3 protein than the adjacent renal tissues (Figure 1J).

**TABLE 1:** The clinic-pathological factors of 93 KIRC patients for survival analysis.

**Clinical characteristic**	**ATF3 expression**		***X*^2^**	***P*-value^*a*^**
	**Up-regulation (*n* = 46)**	**Down-regulation (*n* = 47)**		
**Gender**				
Male	18	32		
Female	28	15	6.7189	<0.001***
**Age**				
≤60	40	21		
>60	6	26	16.584	<0.001***
**Laterality**				
Left	23	32		
Right	23	15	2.4426	0.118
**Tumor size**				
≤4 cm	7	26		
>4 cm	39	21	14.626	<0.001***
**Fuhrman grade**				
G1-2	42	29		
G3-4	4	18	9.7	<0.001***
**Tstage**				
T1-2	36	20		
T3-4	10	27	10.927	<0.001***
**Metastasis**				
No	30	19		
Yes	16	28	4.78	0.028*

^*a*^*p*-value from Chi-square test. *p < 0.05, ***p < 0.001 statistically significant.

Collectively, the above data demonstrated that ATF3 expression was decreased in patients with ccRCC and was negatively associated with advanced tumor stage. This indicated that ATF3 at lower levels might play a key role in the growth and metastasis of ccRCC.

### ATF3 Inhibits ccRCC Cell Proliferation and Colony-Formation, and Leads to Cell Cycle Arrest

**FIGURE 2:**
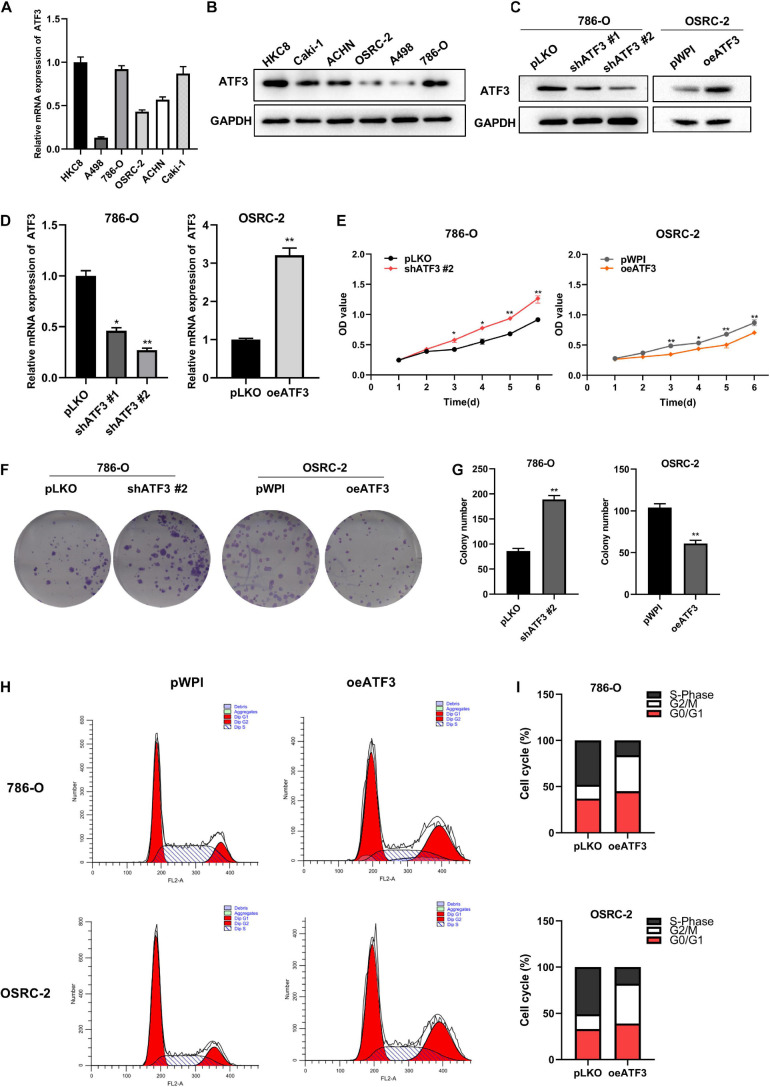
ATF3 inhibits ccRCC cell proliferation, colony-forming ability, and leads to cell cycle arrest at the G2/M checkpoint. **(A)** ATF3 mRNA levels in different ccRCC cell lines compared to those in normal kidney HKC8 cells. **(B)** The protein level of ATF3 in different ccRCC cell lines and normal kidney HKC8 cell line. **(C**,**D)** Knockdown efficiency of shATF3 lentivirus in 786-O cells and overexpressed efficiency of oeATF3 lentivirus in OSRC-2 cells. **(E)** CCK8 assay revealed cell proliferation in 786-O cells and OSRC-2 cells. **(F**,**G)** ATF3 knockdown by shRNA #2 increased colony-forming ability of 786-O cells and overexpression of ATF3 decreased colony-forming ability of OSRC-2 cells. **(F)** Representative images of colonies. **(G)** Statistical analysis of panel **(F)**. **(H,I)** ATF3 overexpression increased cell population in G2/M-phase and decreased cell population in S-phase both in 786-O and OSRC-2 cells as examined by FACS using PI staining. *p < 0.05, **p < 0.01.

To investigate the biological effects of ATF3 in ccRCC cells, we used five ccRCC cell lines and an immortalized human tubule epithelial cell line HKC8 to analyze the mRNA and protein levels of ATF3 through qRT-PCR and WB. All ccRCC cell lines exhibited lower ATF3 expression than the normal kidney cell line HKC8 (Figures 2A,B). We chose 786-O cell, which contains a higher endogenous expression, to knockdown ATF3 using two shRNAs (shATF3#1 and shATF3#2). shATF3#2 was used for further experiments because of its higher knockout efficiency. In addition, we established ATF3 overexpression in OSRC-2 cell, which contains a lower endogenous lower expression (Figures 2C,D). In order to study the effect of ATF3 on proliferation in ccRCC cells, we first used the CCK8 assay to detect the cell growth. The results revealed that knockdown of ATF3 significantly increased the proliferation of 786-O cells and that overexpression of ATF3 inhibited the proliferation of OSRC-2 cells (Figure 2E). Consistently, ATF3 knockdown significantly promoted colony formation in 786-O cells, and ATF3 overexpression suppressed the colony formation ability of OSRC-2 cells (Figures 2F,G). Next, we used FACS analysis to investigate how ATF3 modulates cell cycle progression. The number of cells remaining at different cell cycle phases (G0/G1, S, or G2/M) was quantified as a percentage of the whole for comparison. We found that ATF3 overexpression resulted in a decrease in the percentage of cells in the S phase and in an increase in the percentage of cells in G2/M phase both in 786-O and OSRC-2 cells (Figures 2H,I). Collectively, these results demonstrated that ATF3 reduced the growth of ccRCC cells.

### ATF3 Inhibits Migration and Invasion and Induces Apoptosis in Human ccRCC Cells

**FIGURE 3:**
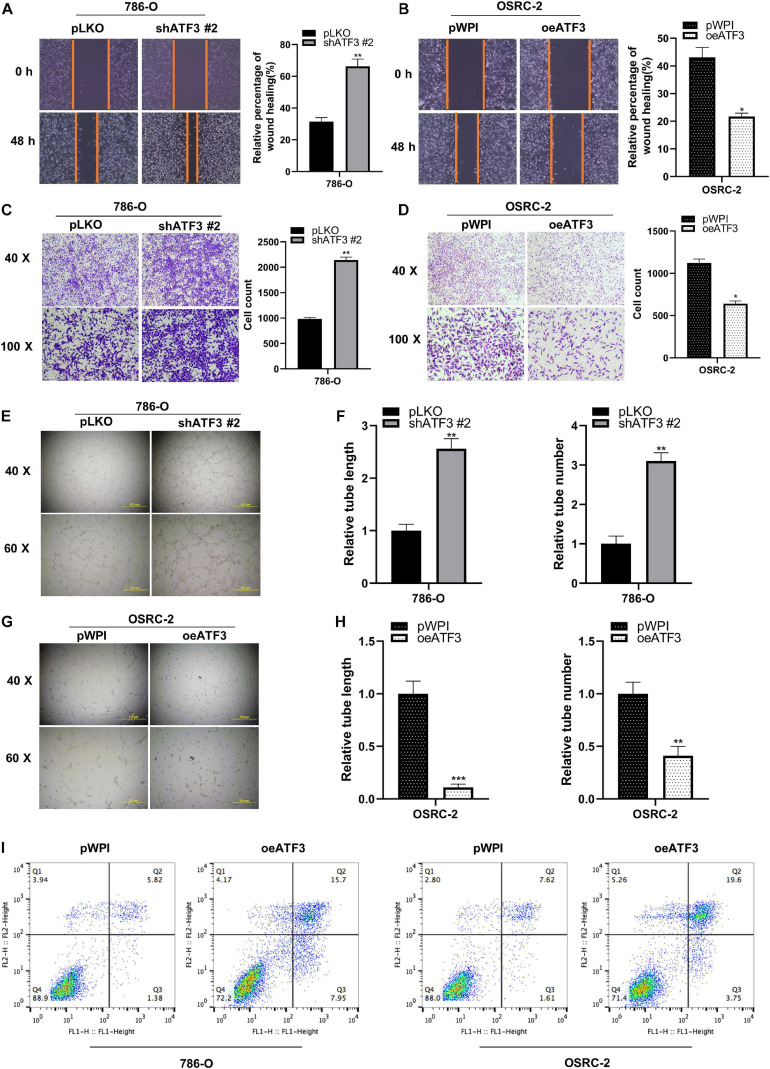
ATF3 inhibits migration, invasion and induced apoptosis in human ccRCC cells. **(A,B)** Wound-healing assay after ATF3 knockdown in 786-O **(A)** and OSRC-2 **(B)** cells. **(C,D)** Invasion assay after ATF3 knockdown in 786-O cells **(C)** and overexpressed in OSRC-2 cells **(D)**. **(E–H)** Vasculogenic mimicry (VM) formation assays were performed using 786-O cells **(E)** transfected with ATF3-shRNA #2 and pLKO, with OSRC-2 cells **(G)** transfected with oeATF3 and pWPI, and the quantitation is on the right of the images **(F,H)**. **(I)** Higher apoptotic index in 786-O and OSRC-2 cells exhibiting overexpression of ATF3 compared to pWPI as detected by FACS. *p < 0.05, **p < 0.01, ***p < 0.001.

To evaluate the potential metastatic function of ATF3 in ccRCC cells, we used 786-O cells to be transfected with shATF3, and the results showed greater migratory capability in the scratch-wound healing model compared to pLKO group (Figure 3A). On the contrary, OSRC-2 cells transfected with oeATF3 showed less motility compared to pWPI group (Figure 3B). Similarly, the effect of ATF3 on ccRCC cell invasion was determined using the transwell invasion assay. The results showed that knocking down ATF3 in 786-O cells increased the cell invasion ability compared to that in the control (pLKO) group (Figure 3C). Overexpression of ATF3 in OSRC-2 cells decreased cell invasion (Figure 3D). The result of vasculogenic mimicry (VM) also confirmed the effect of ATF3 (Figures 3E–H). The apoptosis assay with flow cytometry results demonstrated a higher apoptotic index both in the 786-O and OSRC-2 cells transfected with oeATF3 (Figure 3I). Altogether, these results demonstrated that ATF3 inhibits metastatic potential and induces the apoptosis of ccRCC cells.

### ATF3 Overexpression Suppresses ccRCC Tumor Growth in the *in vivo* Mouse Models

**FIGURE 4:**
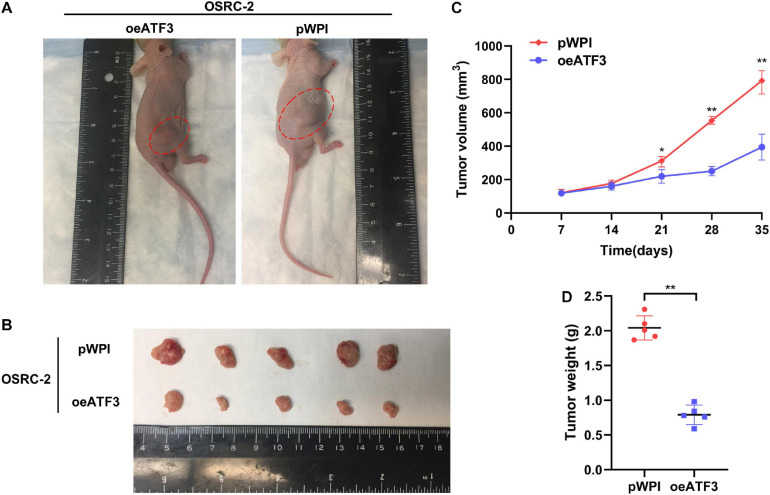
ATF3 overexpression inhibits ccRCC tumor growth. **(A)** Representative images of xenografts (arrows) were taken 5 weeks after injection. **(B)** The gross size of tumors in oeATF3 and control groups. **(C,D)** Analysis of tumor volume **(C)** and weight **(D)** of xenograft tumors. *p < 0.05, **p < 0.01.

We sought to determine whether ATF3 could exert an inhibitory effect on tumor growth *in vivo*. The stabilized oeATF3#2-786-O cells were subcutaneously implanted into nude mice (1 × 10^6^ cells per mouse, five mice per group), and the mice injected with the corresponding empty pWPI vector served as a control group. As expected, the results indicated that the tumor volumes of the oeATF3 group were significantly smaller than those of the pWPI group at 5 weeks (Figures 4A,B). In addition, the result of the linear curve also demonstrated that overexpression of ATF3 significantly suppressed the growth (Figure 4C) and average weight (Figure 4D) of tumors in nude mice. These results suggested that ATF3 could suppress ccRCC tumor growth *in vivo*, which was consistent with the observed results *in vitro*.

### ATF3 Inhibits Epithelial–Mesenchymal Transition (EMT) by Deactivating the β-Catenin Pathway

**FIGURE 5:**
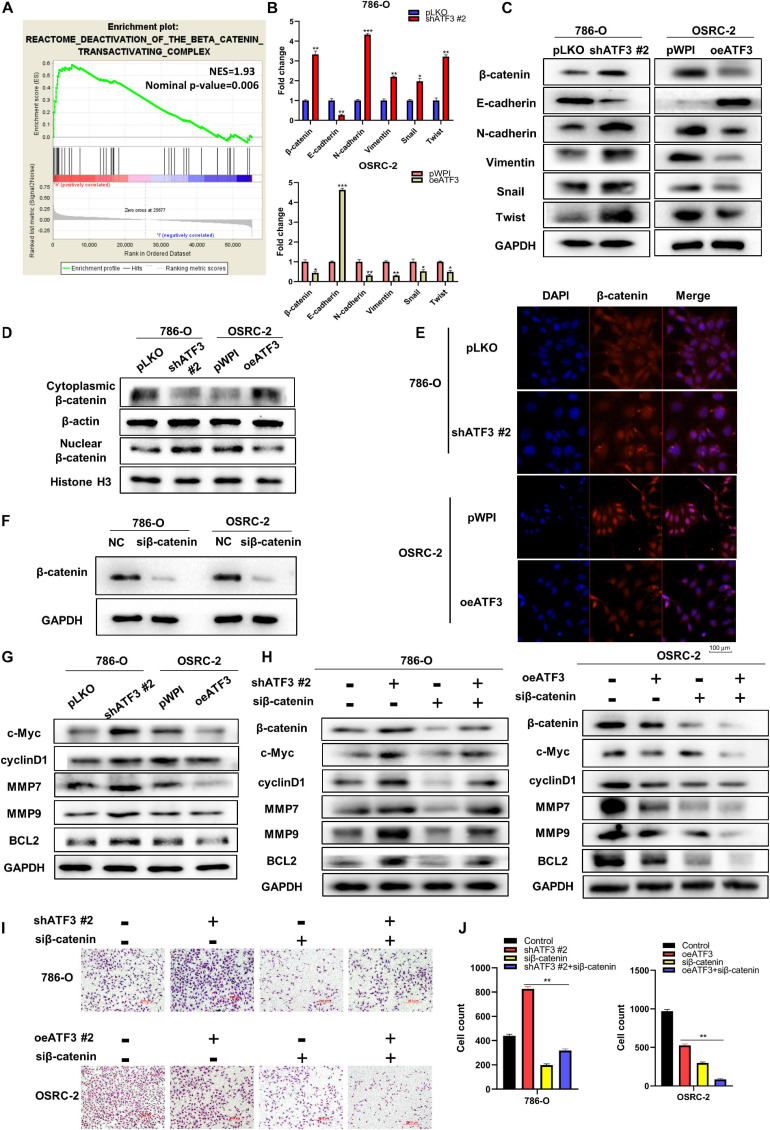
ATF3 inhibits EMT by deactivating the β-catenin pathway. **(A)** GSEA analysis revealed that ATF3 could deactivate the β-catenin transactivating. **(B**,**C)** RT-PCR **(B)** and WB **(C)** assays revealed the expressions of EMT-related markers in ccRCC cells with ATF3 downregulation in 786-O cells and upregulation OSRC-2 cells. **(D,E)** ATF3 inhibits translocation of β-catenin from cytoplasm to nucleus thus reduced the nuclear β-catenin. **(F)** Efficiencies of β-catenin knockdown with siβ-catenin in 786-O and OSRC-2 cells. **(G)** TransWestern blot assays evaluated the expression of downstream target genes of β-catenin, including c-Myc, cyclin D1, mmp7, mmp9, BCL2 in 786-O cells with ATF3 knockdown and OSRC-2 cells with ATF3 overexpression. **(H)** The depletion of β-catenin reversed the protein expression of downstream genes, which were caused by induced ATF3 expression in 786-O cells and enhanced ATF3 expression in OSRC-2 cells. **(I,J)** Invasion phenotype shown that the effect of ATF3 knockdown can be reversed by siβ-catenin in 786-O cells and the effect of ATF3 overexpression can be enhanced by siβ-catenin in OSRC-2 cells **(I)**, and quantitation is on the right of images **(J)**. *p < 0.05, **p < 0.01, ***p < 0.001.

In order to probe the potential molecular mechanisms of ATF3 in ccRCC, we divided the TCGA-KIRC dataset into a high ATF3 expression group and a low ATF3 expression group using the median ATF3 expression as the traction criterion. GSEA was used to compare different biological processes between the two groups. Interestingly, the results of GSEA revealed that ATF3 was strongly associated with the β-catenin transactivating pathway (Figure 5A). β-Catenin is considered to be the central molecule of the Wnt/β-catenin signaling pathway, which also participates in the EMT process. We then used RT-PCR and WB to assess EMT-related markers. The results showed that downregulated ATF3 could significantly suppress the expression of epithelium-related E-cadherin gene, and could dramatically increase the expression of β-catenin and mesenchymal-related markers, such as N-cadherin, vimentin, snail, and twist (Figures 5B,C). The translocation of β-catenin from the cytoplasm to the nucleus leads to the activation of the canonical β-catenin signaling pathway. Therefore, we extracted both nuclear and cytosolic parts from shATF3#2 and oeATF3 cells and their corresponding control cells. The results of WB showed that shATF3#2 decreased the protein expression of β-catenin in the cytoplasm and promoted β-catenin nuclear translocation in 786-O cells. Conversely, when increased the expression of ATF3 with overexpressing ATF3 in the OSRC-2 cells, the expression of β-catenin protein in the cytoplasm was significantly increased, and the β-catenin protein level was obviously decreased in the nucleus (Figure 5D). These results were also confirmed through immunofluorescence assays (Figure 5E). Next, we analyzed the expression of genes downstream of β-catenin to further verify the regulation of β-catenin by ATF3. The expression of cyclin D1, MMP7, MMP9, and BCL2 was increased upon ATF3 depletion in 786-O cells. In contrast, the protein expression of cyclin D1, MMP9, MMP7, and BCL2 was significantly downregulated upon ATF3 overexpression in OSRC-2 cells (Figure 5G). We then detected the effect of β-catenin knockdown using siβ-catenin in both 786-O and OSRC-2 cells (Figure 5F). In addition, β-catenin knockdown, caused by induced ATF3 expression in 786-O cells and enhanced ATF3 expression in OSRC-2 cells, reversed the protein expression of downstream genes compared to that in the control group (Figure 5H). Consistently, we performed invasion assay and obtained the same results (Figures 5I,J). Accordingly, the results indicated that ATF3 regulated EMT progression via the β-catenin-dependent signaling pathway.

### ATF3 Inhibits EMT via Deactivating EGFR/AKT/GSK3β/β-Catenin Signaling in ccRCC Cancer Cells

**FIGURE 6:**
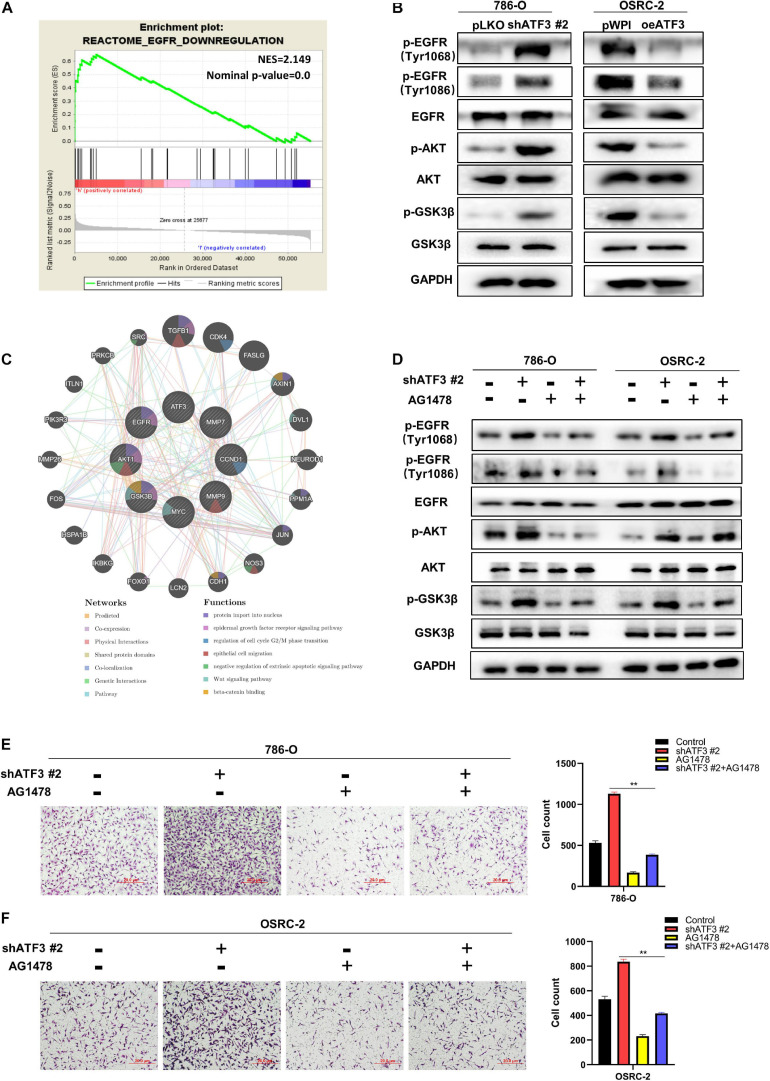
ATF3 inhibits EMT via deactivating EGFR/AKT/GSK3β/β–catenin signaling in ccRCC cancer cells. **(A)** GSEA analyses detected that EGFR-Downregulation was significantly enriched in ATF3 high group. **(B)** Western blot assays identified ATF3 knockdown increased the phosphorylation level of EGFR (at Tyr1068 and Tyr1086), p-AKT and p–GSK3β in 786-O cells, while ATF3 overexpression has the opposite result in OSRC-2 cells. **(C)** GeneMANIA database shown the research genes enhanced on the relevant function of protein import in nucleus, epidermal growth factor receptor signaling pathway, regulation of cell cycle G2/M phase transition, epithelial cell migration, negative regulation of extrinsic apoptotic signaling pathway, Wnt signaling pathway, beta-catenin binding. **(D)** Western blot assays detect the phosphorylation levels of EGFR, AKT and GSK3β treated with EGFR specific inhibitor (AG1478) after ATF3 knockdown. **(E,F)** The enhanced effect of invasion by knockdown ATF3 also can be reversed by AG1478 in 786-O cells **(E)** and OSRC-2 cells **(F)**. **p < 0.01.

To investigate how ATF3 reduces β-catenin signaling, we conducted GSEA analysis. Our result showed that canonical WNT signaling was not tightly associated with ATF3 expression (data not shown). Unexpectedly, we found that EGFR signaling, one non-canonical pathway bears the capacity to activate β-catenin via AKT/GSK3β (Hofmockel et al., 1997; Hashmi et al., 2018; Ge et al., 2020), was significantly enriched in the ccRCC patients with low ATF3 expression (Figure 6A), suggesting loss of ATF3 may activate β-catenin via EGFR/AKT/GSK3β signaling. As observed in Figure 6B, WB results revealed that ATF3 knockdown increased the phosphorylation level of EGFR (at Tyr1068 and Tyr1086), p-AKT, and p−GSK3β in 786-O cells, whereas ATF3 overexpression had the opposite effect in OSRC-2 cells. These findings were consistent with our speculation that upregulation of β-catenin by shATF3 was due to the activation of EGFR/AKT/GSK3β signaling. To confirm the cross-talk between ATF3 and EGFR signaling, we performed interruption assay using EGFR inhibitor. Indeed, the increased p-EGFR, p-AKT, and p-GSK3β levels by shATF3#2 could be reversed by adding the inhibitor of EGFR-specific tyrosine kinase (AG1478, 10 μM) (Figure 6D). In addition, the enhanced effect on cell proliferation (Supplementary Figures 2A,B) and cell invasion (Figures 6E,F) observed upon knockdown of ATF3 was also reversed by AG1478 in 786-O and OSRC-2 cells. To further confirm the participation of EGFR/AKT/GSK3β into shATF3 promoted ccRCC progression, we applied AKT inhibitor or GSK3β inhibitor to examine whether it can block the biological functionalities of ATF3. As expected, inhibition of AKT by MK-2206 (1 μM) could blunt shATF3-induced β-catenin, cell proliferation and cell invasion of both 786-O and OSRC-2 cells (Supplementary Figure 3). Similar results were gained when GSK3β inhibitor BIO (1 μM) was used (Supplementary Figure 4). Thus, these data suggest that activation of β−catenin by loss of ATF3 was dependent of EGFR/AKT/GSK3β signaling, which probably contributes to ccRCC development.

## Discussion

Currently, ccRCC remains the most common cancer (Hsieh et al., 2017). Although current treatments benefit patients, the persistence of metastatic ccRCC is still quite high (Verhoest et al., 2009; Jonasch et al., 2014). Therefore, there is an urgent need to identify novel molecules that represent pivotal biomarkers of ccRCC, in order to develop new therapeutic strategy for better treatment of ccRCC patients.

ATF3 may perform different functions in various tumors. In hepatocellular carcinoma and esophageal squamous cell carcinoma, ATF3 expression was lower than that in normal adjacent tissues and could suppress tumor growth (Xiaoyan et al., 2014; Li et al., 2016). However, in lung cancer, the upregulated ATF3 promotes cancer cell proliferation, invasion, and migration (Li et al., 2017). ATF3 displays conflicting functions in various diseases, and this may be due to the complicated tumor microenvironment, such as the community of genomically altered non-neoplastic cells, cancer cells, and various microorganisms. In this study, our clinical pathological and TCGA data analysis of ccRCC demonstrated that ATF3 was significantly downregulated in tumors compared to in adjacent non-tumor tissues. Moreover, ATF3 was positively correlated with the OS and DFS of ccRCC patients. Our study further validated that ATF3 could inhibit ccRCC cell proliferation, colony formation, and metastasis both *in vitro* and *in vivo*. Besides, GEPIA web tools also showed that ATF3 was significantly expressed with poor prognosis in many cancers, further suggesting that ATF3 might play an anti-oncogenic role in various types of human cancer.

According to the GESA of a single gene, ATF3 could deactivate β-catenin transactivation. Activation of the β-catenin signaling pathway by β-catenin nuclear translocation plays a significant role in EMT process. The disturbance of this signaling pathway leads to a change in many biological processes, including cell autophagy (Romero et al., 2018), cell migration (Ji et al., 2013), and cell apoptosis (Rosenbluh et al., 2012). Our study demonstrated that ATF3 overexpression could decrease the protein expression of β-catenin and reduce the translocation of β-catenin into the nucleus. It has been reported that the downstream proteins of β-catenin are closely related to cell functions, such as c-Myc and cyclin D1 which are related to cell proliferation, MMP7 and MMP9 which are related to cell invasiveness, and BCL2 which is involved in anti-apoptosis (Li et al., 2020). Herein, our research proved that ATF3 knockdown enhanced the expression of c-Myc, cyclin D1, MMP7, MMP9, and BCL2, while ATF3 overexpression showed the opposite effect. Finally, many biological processes can be regulated in kidney cancer cells by influencing these genes.

Deep mechanistic exploration demonstrated that ATF3-reduced β-catenin was attributable to the downregulation of EGFR signaling pathway. According to some reports, EGFR is associated with most solid tumors (Goss et al., 2016), and is positively correlated with the clinical histopathological characteristics of ccRCC patients (Pu et al., 2009). A previous study reported that the activation of the EGFR/AKT/β-catenin axis promoted EMT in pancreatic cancer cells (Ge et al., 2020). Interestingly, our data also suggested that ATF3 reduced the phosphorylation of EGFR, deactivating the phosphorylation of AKT and its downstream molecule GSK3β, thereby downregulating the expression of β−catenin to decrease invasive motility and suppress EMT, which was consistent to previous report. And deactivation of EGFR, AKT or GSK3β with its corresponding inhibitor could block shATF3 induced β−catenin expression, cell proliferation and cell invasion in ccRCC cells, suggesting they formed as one central signaling axis to promote ccRCC development.

## Conclusion

Overall, our study illustrates that ATF3 suppresses EMT by deactivating EGFR/β-catenin signaling in ccRCC cells. Furthermore, the findings suggest that ATF3 may be a potential biomarker for kidney cancer diagnosis and prognosis.

## Data Availability Statement

The raw data supporting the conclusions of this article will be made available by the authors, without undue reservation.

## Ethics Statement

The animal study was reviewed and approved by Institutional Animal Use and Care Committee of Nanjing Medical University.

## Author Contributions

LZ, XQ, LFZ, and SG conceived and designed the study. SG, XQ, LG, CY, LFZ, XS, SZW, and SW contributed to the conduction of the experiments. LG and SW contributed to data analysis. CY, LFZ, and XS provided clinical samples and clinical information. SG wrote the manuscript. LZ, XQ, and LG supervised the research. All authors read and approved the final manuscript.

## Conflict of Interest

The authors declare that the research was conducted in the absence of any commercial or financial relationships that could be construed as a potential conflict of interest.

## Abbreviations

ccRCC: clear cell renal cell carcinoma
ATF3: activation transcription factor 3
TCGA: The Cancer Genome Atlas
CCK8: cell counting kit-8
shRNA: short hairpin RNA
PBS: phosphate-buffered saline
CyclinD1: cyclin-dependent kinase 1
PVDF: polyvinylidene fluoride
CI: confidence interval
SD: standard deviation
HR: hazard ratio
ANOVA: analysis of variance
DMEM: Dulbecco’s modified Eagle medium
EMT: epithelial–mesenchymal transition
IHC: immunohistochemistry
qRT-PCR: quantitative reverse transcription polymerase chain reaction
TBST: Tris-buffered saline and Tween 20
SDS: sodium dodecyl sulfate.
