# Supplementary material for: ATF3 Suppresses Growth and Metastasis of Clear Cell Renal Cell Carcinoma by Deactivating EGFR/AKT/GSK3β/β-Catenin Signaling Pathway
Source: Front Cell Dev Biol. 2021 Mar 19;9:618987. doi: 10.3389/fcell.2021.618987 (PMC8017234; doi:10.3389/fcell.2021.618987)
Supplement: Supplementary file 1 [file Table_1.DOCX]

**Supplementary data**

# **ATF3 Suppresses Growth and Metastasis of Clear Cell Renal Cell Carcinoma via Deactivating** **EGFR/AKT/ GSK3β /β‐catenin Signaling Pathway**

Shenglin Gao^1, #^, Lei Gao^2, #^, Simin Wang^3, #^, Xiaokai Shi^1^, Chuang Yue^1^, Shuzhang Wei^1^, Li Zuo^1, *^, Lifeng Zhang^1, *^, Xihu Qin^4, *^

**Institution:**

^1^ Department of Urology, The Affiliated Changzhou No. 2 People's Hospital of Nanjing Medical University, Changzhou, Jiangsu, 213000, China

^2^ Department of Urology, The Second Hospital of Hebei Medical University, Shijiazhuang, Hebei, 050000, China

^3^ Changzhou Third People's Hospital, Changzhou, Jiangsu, 213000, China

^4^ Department of General Surgery, The Affiliated Changzhou No. 2 People's Hospital of Nanjing Medical University, Changzhou, Jiangsu Province, China, Jiangsu, 213000, China

^#^ These authors contributed equally to this work.

^*^Correspondence to:

**Name:** Li Zuo (first corresponding author)

**Address:** Department of Urology, The Affiliated Changzhou No. 2 People's Hospital of Nanjing Medical University, Changzhou, Jiangsu, China

**Email:** [zuoli@njmu.edu.cn](mailto:zuoli@njmu.edu.cn)

**Name:** Lifeng Zhang

**Address:** Department of Urology, The Affiliated Changzhou No. 2 People's Hospital of Nanjing Medical University, Changzhou, Jiangsu, China

**Email:** nj-likky@163.com

**Name:** Xihu Qin

**Address:** Department of General Surgery, The Affiliated Changzhou No. 2 People's Hospital of Nanjing Medical University, Changzhou, Jiangsu Province, China, Jiangsu, 213000, China

**Email:** [qinxihu@126.com](mailto:qinxihu@126.com)

**Running title:** ATF3 suppresses progression of clear cell renal cell carcinoma via EGFR/β-catenin pathway

**Keywords:** Clear cell renal cell carcinoma; Activation transcription factor 3; growth; metastasis; GSEA

**Table S1 Sequences of primers used for experiments in this study**

| **Name** | **Primer Sequence (5’-3’)** |
| --- | --- |
| GAPDH-F | ACAACTTTGGTATCGTGGAAG |
| GAPDH-R | ACAACTTTGGTATCGTGGAAG |
| ATF3-F | TTTGCTAACCTGACGCCCTT |
| ATF3-R | TGACTGATTCCAGCGCAGAG |
| β-catenin-F | AAAGCGGCTGTTAGTCACTGG |
| β-catenin-R | CGAGTCATTGCATACTGTCCAT |
| E-cadherin-F | ATTTTTCCCTCGACACCCGAT |
| E-cadherin-R | TCCCAGGCGTAGACCAAGA |
| N-cadherin-F | GAGCATGCCAAGTTCCTGAT |
| N-cadherin-R | TGGCCACTGTGCTTACTGAA |
| Vimentin-F | AGTCCACTGAGTACCGGAGAC |
| Vimentin-R | CATTTCACGCATCTGGCGTTC |
| Snail-F | ACCACTATGCCGCGCTCTT |
| Snail-R | GGTCGTAGGGCTGCTGGAA |
| Twist-F | GAGTCCGCAGTCTTACGAGG |
| Twist-R | CTGCCCGTCTGGGAATCACT |

**Table S2 The antibodies for the Western blotting and IHC**

| **Antibody** | **Source** | **Catalogue number** | **Dilution** | **Application** | **Company** |
| --- | --- | --- | --- | --- | --- |
| ATF3 | Rabbit | ab200655 | 1:1000 | WB | Abcam |
| β-catenin | Rabbit | ab32572 | 1:1000 | WB | CST |
| GAPDH | Mouse | sc-166574 | 1:5000 | WB | Santa Cruz |
| Cyclin D1 | Mouse | sc-8396 | 1:1000 | WB | Santa Cruz |
| c-Myc | Mouse | sc-40 | 1:1000 | WB | Santa Cruz |
| Histone H3 | Mouse | ab4076 | 1:1000 | WB | Abcam |
| MMP7 | Mouse | sc-80205 | 1:1000 | WB | Santa Cruz |
| MMP9 | Rabbit | ab38898 | 1:1000 | WB | Abcam |
| BCL2 | Mouse | sc-7382 | 1:1000 | WB | Santa Cruz |
| E-cadherin | Mouse | sc-8426 | 1:1000 | WB | Santa Cruz |
| N-cadherin | Mouse | sc-59987 | 1:1000 | WB | Santa Cruz |
| Snail | Mouse | sc-271977 | 1:1000 | WB | Santa Cruz |
| Twist | Rabbit | 127310 | 1:1000 | WB | Gene Tex |
| p-AKT | Rabbit | #4060 | 1:2000 | WB | CST |
| AKT | Rabbit | #4691 | 1:1000 | WB | CST |
| p-EGFR  (Tyr1068) | Rabbit | #2234 | 1:1000 | WB | CST |
| p-EGFR  (Tyr1086) | Rabbit | #2220 | 1:1000 | WB | CST |
| EGFR | Rabbit | #2232 | 1:1000 | WB | CST |
| p‐GSK3β | Rabbit | #9323 | 1:1000 | WB | CST |
| GSK3β | Rabbit | #12456 | 1:1000 | WB | CST |
| β-actin | Mouse | sc-47778 | 1:1000 | WB | Santa Cruz |
| ATF3 | Mouse | ab191513 | 1:25 | IHC | Abcam |

**Supplementary Figure1**


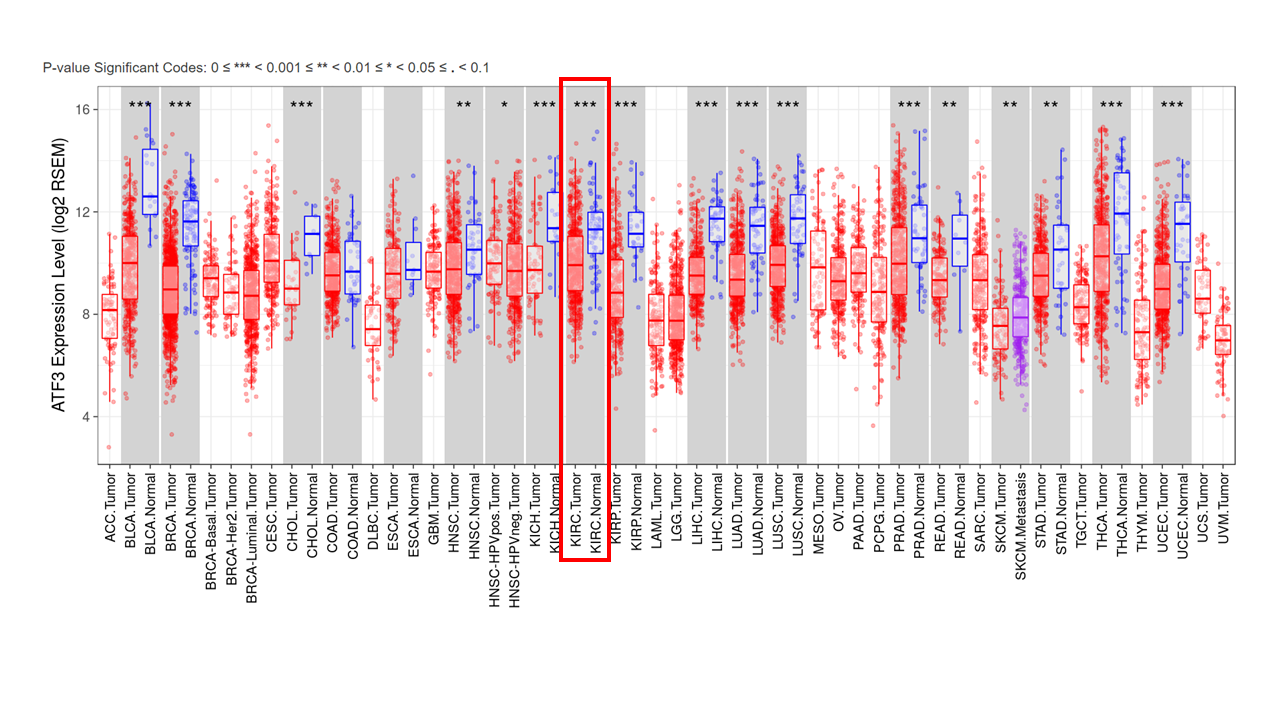


TIMER webtools analysis that across 13 human tumor types (BLCA, BRCA, CHOL, HNSC, KICH, KIRC, KIRP, LIHC, LUAD, LUSC, PRAD, READ, SKCM) tumor(red) had significantly lower ATF3 expression compared to paired normal (blue) samples.
