# Supplementary figures and images for: ATF3 Suppresses Growth and Metastasis of Clear Cell Renal Cell Carcinoma by Deactivating EGFR/AKT/GSK3β/β-Catenin Signaling Pathway
Source: Front Cell Dev Biol. 2021 Mar 19;9:618987. doi: 10.3389/fcell.2021.618987 (PMC8017234; doi:10.3389/fcell.2021.618987)

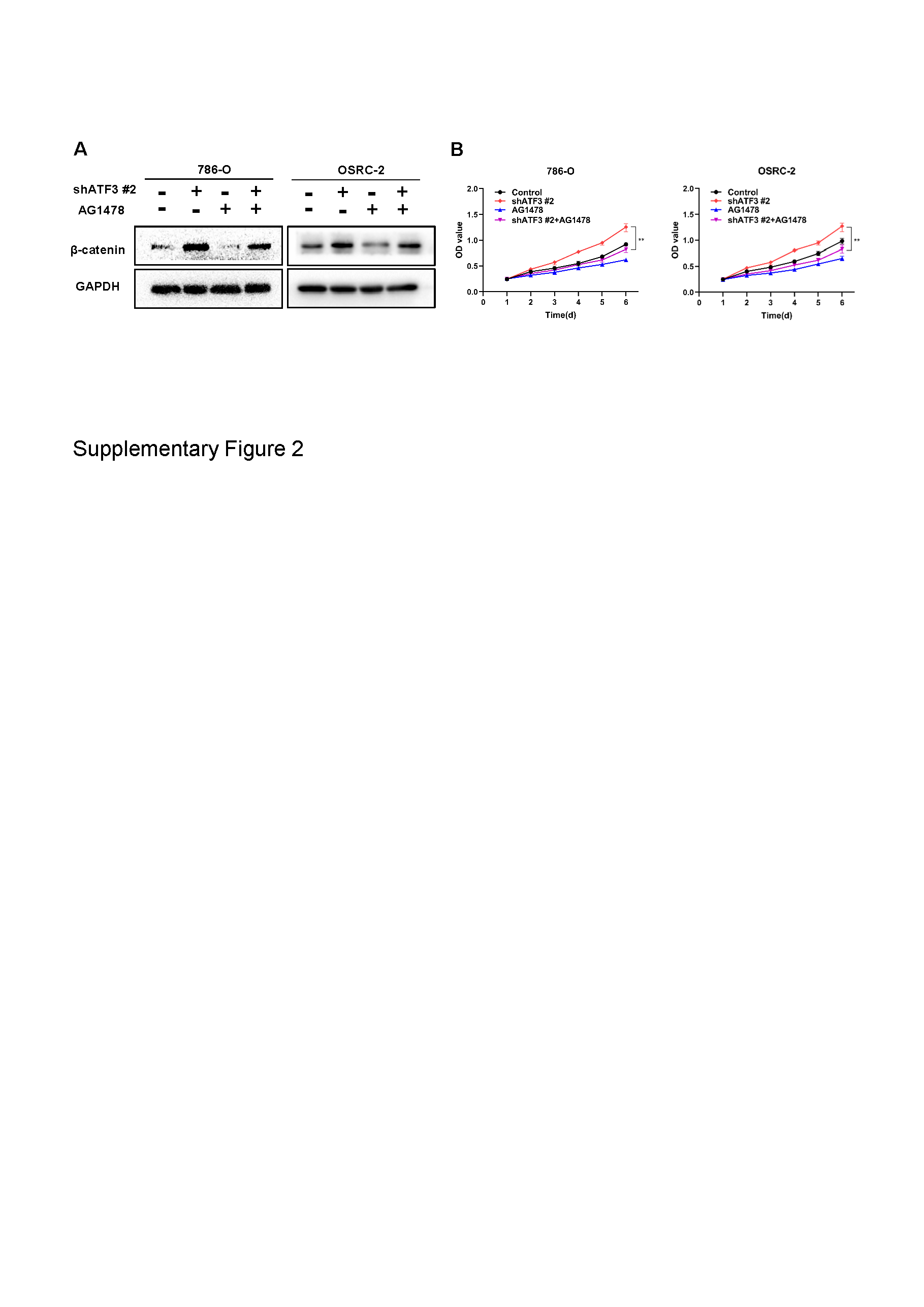

Supplement: Supplementary file 2 [file Image_1.TIFF]

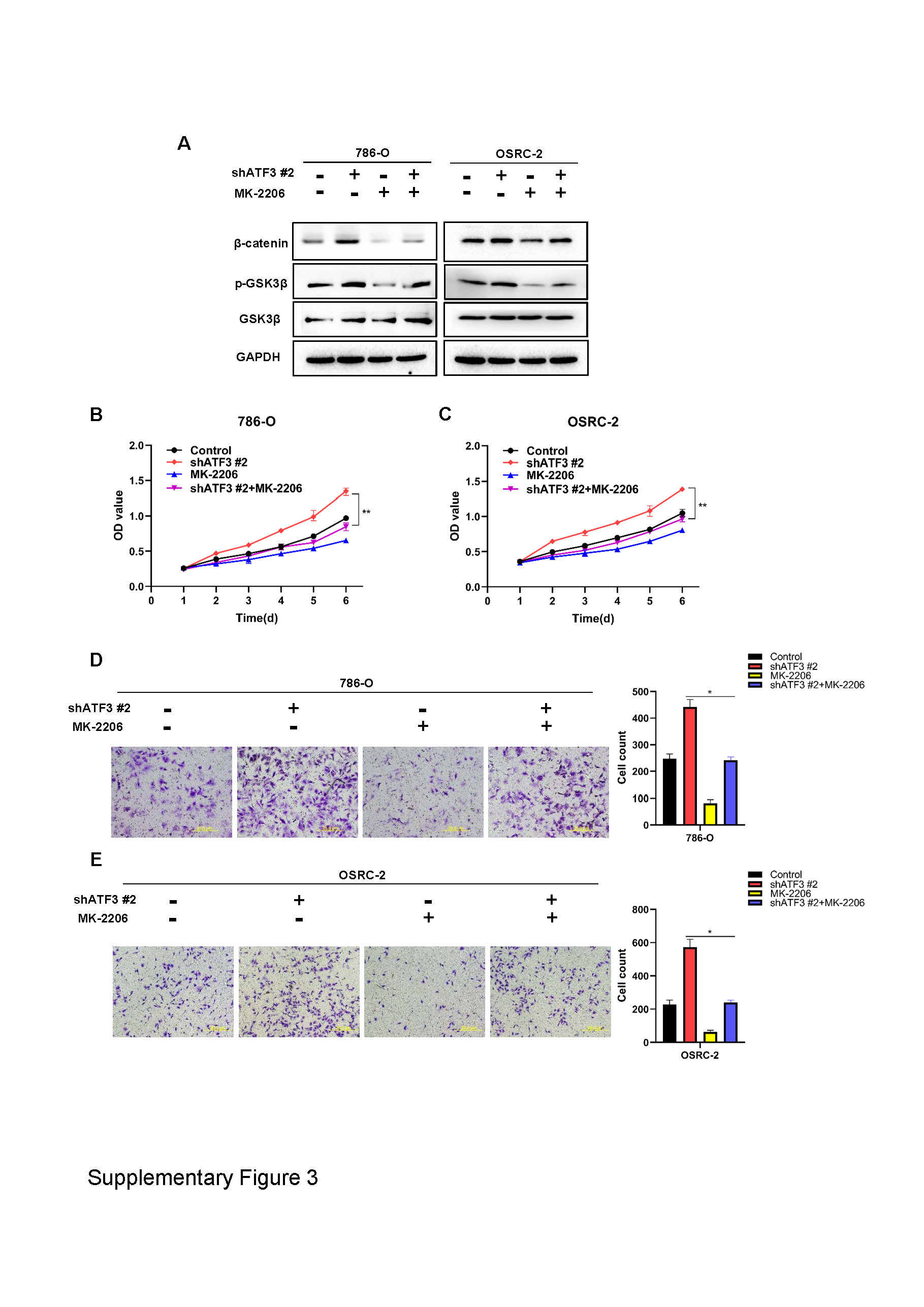

Supplement: Supplementary file 3 [file Image_2.TIFF]

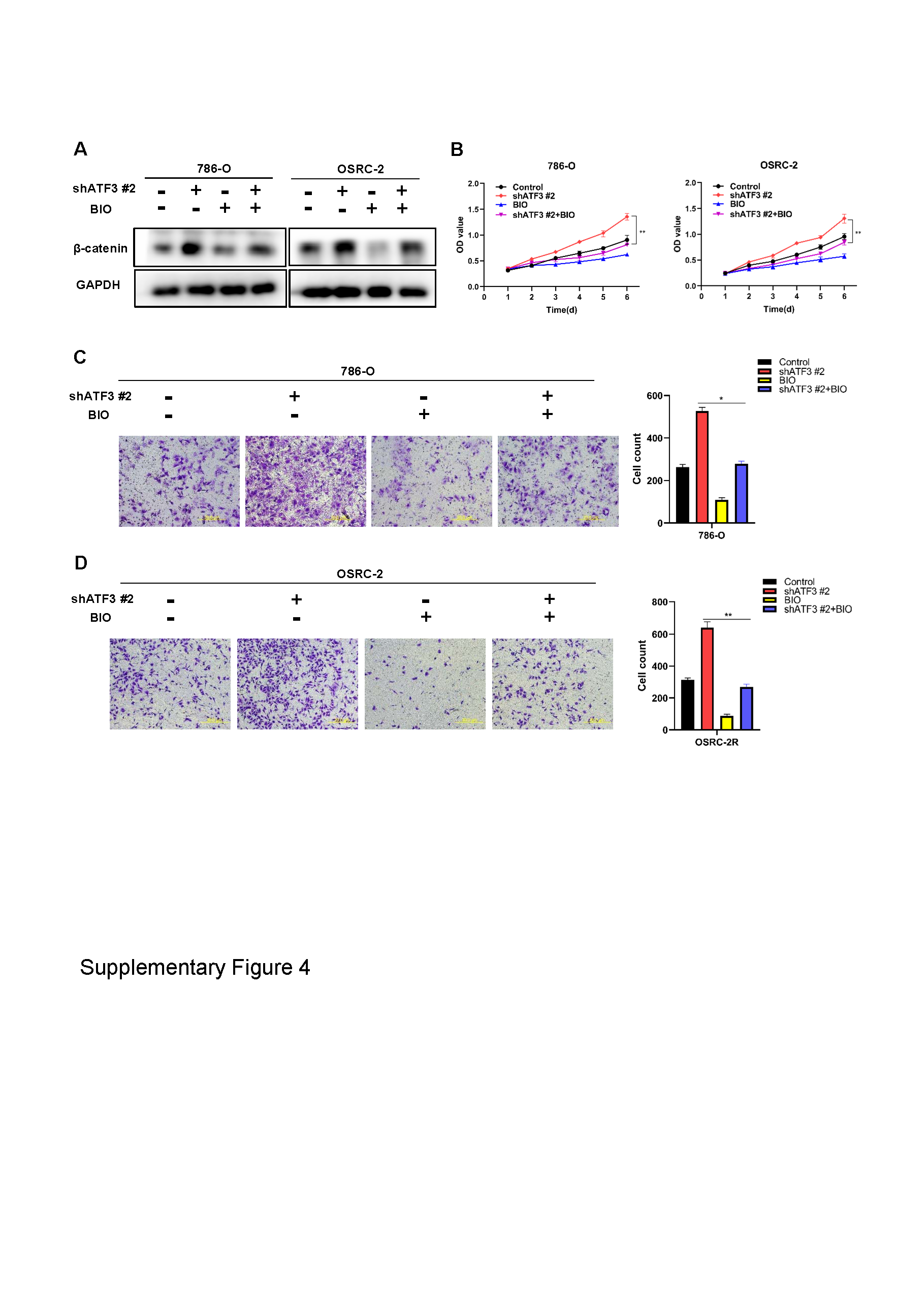

Supplement: Supplementary file 4 [file Image_3.TIFF]
